# Natural language processing models for predicting treatment outcomes in internet-delivered cognitive behavioral therapy

**DOI:** 10.1016/j.invent.2025.100879

**Published:** 2025-10-10

**Authors:** Nils Hentati Isacsson, Lucía Gómez-Zaragozá, Fehmi Ben Abdesslem, Magnus Boman, Viktor Kaldo

**Affiliations:** a Centre for Psychiatry Research, Department of Clinical Neuroscience, Karolinska Institutet, & Stockholm Health Care Services, Region Stockholm, Sweden; b HUMAN-Tech Institute, Universitat Politècnica de València, Valencia, Spain; c Department of Computer Science, RISE Research Institutes of Sweden, Stockholm, Sweden; d Division of Psychiatry, University College London, UK; e Department of Medicine Solna, Clinical Epidemiology Division, Karolinska Institutet, Stockholm, Sweden; f Department of Psychology, Faculty of Health and Life Sciences, Linnaeus University, Växjö, Sweden

## Abstract

**Objective:**

Predicting treatment outcome has the potential to enhance Internet-delivered Cognitive Behavioral Therapy (ICBT). One aspect of guided ICBT is patient-therapist interaction through written messages. With Natural language processing (NLP) these could be leveraged to predict outcome; however current evidence is limited. This study investigates the predictive accuracy of NLP models for treatment outcomes and evaluates whether NLP provides additional predictive value beyond symptom variables.

**Methods:**

Patient-therapist messages from 6613 patients undergoing 12 weeks of treatment were used to train three types of NLP models: Term Frequency-Inverse Document Frequency (TF-IDF), Bidirectional Encoder Representations from transformers (BERT), and BERT for Longer Text (BELT). These were trained both with and without symptom variables from the initial treatment period to predict post-treatment symptoms. A dummy model was also used, and a linear regression model acted as a symptoms only benchmark. Multiple imputation addressed missing data, and nested cross-validation was used.

**Results:**

The symptom only model performed best. Only BERT outperformed the dummy model, achieving a Root Mean Squared Error (RMSE) of 0.17 compared to RMSE of 0.18. Adding symptom variables to the BERT model significantly increased its accuracy, but not the RMSE metric. The best linear regression benchmark based on symptoms only had a BACC of 70 % (F_1_-score of 0.66) which outperformed the BERT model with 60 % (F_1_: 0.55) and the combined BERT plus symptoms model achieved 68 % (F_1_: 0.62).

**Conclusion:**

These initial findings indicate a small predictive value from patient-therapist written message interaction but added no value beyond using only symptoms to predict post-treatment symptoms. Further research is needed to refine NLP-methods for use in psychological treatment, and more accurately assess the predictive potential of text-based interactions during ICBT.

## Highlights

• Early prediction of patients at risk of not benefitting enough in ICBT can help adapt treatment and allocate resources
• Including text from therapist-patient conversations from the initial treatment period gives a small predictive signal
• Adding self-rated symptoms to NLP models improves predictive accuracies, but are not superiour to models only using symptoms
• NLP models have many potential uses, but current use for predictions of treatment outcome are limited.

## 1 Introduction

Mental health problems pose a significant global burden, yet many individuals who need support lack access to care (Kessler et al., 2009). During the last two decades, Internet-delivered Cognitive Behavioral Therapy (ICBT) has been increasingly adopted in regular clinical practice (Titov et al., 2018). ICBT offers the advantage of delivering effective treatment to a broader population at a lower cost compared to face-to-face therapy and has been shown to have comparable outcomes to in-person CBT (Andersson et al., 2019). However, many patients (30–60 %) do not benefit enough from ICBT (Rozental et al., 2019), underscoring the need for more personalized treatment approaches.

Predicting treatment outcomes holds promise for individual treatment adaption (Forsell et al., 2019; Hentati Isacsson et al., 2024a; Hentati Isacsson et al., 2024b; Hornstein et al., 2023), with initial use of predictions in trials showing promising effects from such adaptions (Forsell et al., 2019). Predicting treatment outcome during the early stages of the treatment period enhances the predictive accuracy (Hentati Isacsson et al., 2024a) but predictions cannot occur too late or the possibilities to adapt treatment will be limited. Prediction models using only symptoms can reach a hypothesized clinical useful accuracy of 67 % (Forsell et al., 2022; Hentati Isacsson et al., 2024a). Despite these predictive models, many overlook the key element of therapist-patient communication in guided therapy, leaving the potential of using these text exchanges to predict outcomes largely unknown. However, ICBT with its structured format and reliance on text-based communication, presents a unique opportunity to build a substantial text corpus for analysis, unlike the arduous process of transcripts from face-to-face therapy. Furthermore, manually created categories from text-communication in ICBT correlates with symptom outcome (Holländare et al., 2016) indicating a possible predictive value.

In the field of Natural language processing (NLP) several approaches have been used to extract useful information regarding mental health treatment and diagnoses (Glaz et al., 2021). NLP can be used for several goals from text analysis and understanding, text generation to text classification or prediction (Tunstall et al., 2022). Despite this, a recent review found that most of the literature of NLP in mental health does not concern using text for predicting treatment outcome, but rather on predicting diagnose, categorizing texts (emotional content, or therapeutic processes) and evaluating links to concurrent symptoms (Glaz et al., 2021). In the review of Glaz et al., only three out of 102 studies prospectively predicted symptom outcomes after treatment using patient-therapist text interactions.

The most common approach was applying NLP techniques to categorize patient text (e.g., emotional content or therapy-process related), which was then used in prediction models. For example, (Burkhardt et al., 2022) used a pretrained BERT model to classify patient messages into emotional categories, achieving an F_1_ score of 0.52 in a sample of 6500. In contrast, (Gogoulou et al., 2021) found that the text representation using Term Frequency-Inverse Document Frequency (TF-IDF) outperformed word-embeddings in predicting treatment outcomes from patient homework texts in ICBT, with an F_1_ score of 0.69 in a sample of 1986 depressed patients. Lastly, (Hoogendoorn et al., 2017) combined various text categorizations (e.g., sentiment, topic, word usage) with baseline data to predict treatment outcomes in ICBT for social anxiety disorder, achieving an F_1_ score of 0.73, though the small sample size of 69 limited generalizability, and baseline data could either improve or reduce model accuracy.

Overall, recent trends indicate that the most popular large language models (LLM) for NLP methods in mental health, are based on the transformer architecture of BERT (Guo et al., 2024). BERT models are particularly well-suited for text understanding and prediction due to their bidirectional nature and ability to be fine-tuned for specific prediction tasks. In contrast, while research into generative LLMs, such as GPT (Generative Pre-trained Transformer; OpenAI et al., 2024), is also widespread, these models are primarily focused on text generation rather than prediction tasks.

Thus, while previous research suggests that text-based data can be used to predict treatment outcomes within psychological treatment, the overall accuracy of these models remains unclear, especially when used prospectively. Additionally, the relative predictive accuracy of using text data compared to symptoms alone is difficult to assess, and the potential synergistic effect of combining both has largely been unexplored.

## 2 Objectives

The aim of this paper is to evaluate predictive models using text-based interactions between patients and therapists in predicting symptom outcomes in ICBT. Specifically, we compare: (1) the predictive performance of NLP models that rely solely on text input and (2) the added value of incorporating text-derived predictions alongside traditional symptom variables to improve outcome prediction. Text data from patient-therapist interaction and symptom data will be included up to week 5 in treatment since the aim is to test the predictive accuracy while there is time for treatment adaptions (Forsell et al., 2019; Hentati Isacsson et al., 2024a).

## 3 Methods

The study is a prospective prediction study using observational data, including the communication between therapist and patient, from a regular care clinic to predict individual symptom outcomes and compare prediction models. Ethical approval was received from the regional ethical review board in Stockholm (Dnr: 2011/2091-31/3, amendment 2016/21-32, 2017/2320-32, and 2018/2550-32).

### 3.1 Setting & participants

The participants (*n* = 6613) were routine care patients at the Internet psychiatry clinic in Stockholm, Sweden between January 2008 and January 2020 (Titov et al., 2018). They received 12 weeks of ICBT for either major depressive disorder (*n* = 3038), panic disorder (*n* = 1740) or social anxiety disorder (*n* = 1835). The treatments have shown positive results, are well established and are guided by a licensed clinical psychologist (El Alaoui et al., 2015; Hedman et al., 2013, Hedman et al., 2014).

Each treatment consists of condition-specific established CBT techniques, including different types of exercises, and weekly self-assessments of the primary symptoms. The text-based messages between therapist and patient encompass both open communication and the patient's responses to homework questions.

### 3.2 Symptom data

The primary continuous outcome for each treatment, were: Montgomery-Åsberg Depression Rating Scale-Self report (MADRS-S) (Montgomery and Asberg, 1979) for major depressive disorder, Panic Disorder Symptom Scale-Self Report (PDSS-SR) (Houck et al., 2002) for panic disorder, and Leibowitz Social Anxiety Scale-Self Report version (LSAS-SR) (Fresco et al., 2001) for social anxiety disorder. Assessments were conducted at screening, before treatment start, on a weekly basis during treatment, and after treatment. In the models using symptom data the symptoms from screening and up to and including week 4 (day 28) in treatment were used.

We pooled all treatment data into one dataset since it is beneficial for developing predictions models for both symptom outcomes (Hentati Isacsson et al., 2024a) as well as dropout prediction (Zantvoort et al., 2024). Models created with a pooled dataset from the same context show an improved predictive performance compared to individual models for each treatment group, possibly due to increasing the sample size and utilizing more robust predictive patterns (Hentati Isacsson et al., 2024a; Zantvoort et al., 2024). To account for different scales of the symptom questionnaires a min-max transformation (Cohen et al., 1999) based on the questionnaire min and max, was applied to each intervention individually. Min was 0 for all three questionnaires and max was: 28, 54, 144 for PDSS-SR, MADRS-S, LSAS-SR, respectively. To evaluate and compare models using both continuous performance measures as well as binary performance measures, the continuous prediction was dichotomized in retrospect. This dichotomization was made based on if the symptom score was either below the designated cut-off for the symptom questionnaire or reflected a 50 % reduction in symptoms. See symptom outcome in supplement for a full description.

In the models using symptom data as extra predictors, information about which treatment the patient was in were also included.

### 3.3 Text data

The 6613 patients had a total of 182,698 messages between themselves and their therapists, including the text-based homework answers sent from the patients. Because of the timepoint of prediction only messages before day 29 were used, resulting in 68,094 messages, and message text was aggregated per patient (from both therapist and patient). This was done to help capture the therapeutic context between patient and therapist, since the therapists' text are also expective to be predictive, an exchange which overall was aimed to inform the prediction. The texts had been cleaned from repetitive automated informative text sent from the treatment program (e.g. ‘Hi welcome to the treatment’) and furthermore homework responses which contained formatting code entries was stripped so the question and answered remained. For the bag-of-words approach using TF-IDF punctuations and stop words were also removed, and the text was then lemmatized using Spacy in Python (Honnibal and Montani, 2017).

For descriptive stats on number of words in messages the top 5 % quantile of number of words sent were removed due to the heavy outlier influence this had on the descriptives statistics.

### 3.4 Prediction models

The following models were used for analyses: A dummy regressor (DR) only predicting the mean of the outcome, Linear regression (LR), a Term Frequency-Inverse Document Frequency (TF-IDF) (Sparck Jones, 1972) representation using Elastic Net regression (EN), a Bidirectional Encoder Representations from transformers (BERT) model (Devlin et al., 2019), and a modified framework for BERT called BERT for Longer Text (BELT) (BERT For Longer Text, 2022/, 2024). All models were implemented in Python 3.10.12 (Python Software Foundation, 2023) using scikit-learn (Pedregosa et al., 2011), and PyTorch (Paszke et al., 2019).

The DR predicts only the mean of the outcome and represent a null model. The LR uses only the symptom variables together with treatment indicator. LR are considered the benchmark method representing the predictive capabilities of a simple model, with easily accessible variables.

TF-IDF using Elastic Net regression (EN) is considered the baseline NLP model. TF-IDF is a bag-of-word model meaning it counts the occurrences of each word in the texts and rescale the word occurrences based upon the relative occurrences across all the texts. This creates a sparse matrix with weighted scores for each word occurrence for patients. To reduce the size of the matrix and increase predictive power words used by more than 80 % of texts are discarded.

The BERT model combines both text representation and prediction training (in comparison to TF-IDF which is combined with Elastic Net Regression) and our model is pre-trained on a large Swedish dataset (“bert-base-swedish-cased” from Huggingface's repository) (Hugging Face, 2022) and subsequently fine-tuned on our dataset. Each text is represented by tokens which are units of text that the model processes. Tokens are segments of text which could be whole words, or word segments. BERT is limited by only being able to consider text sequences up to 510 tokens, because of this the sequence of words for each patient was truncated to consider the first 128 tokens sent and the last 382 sent (within the 28 days), to better capture the entire message sequence.

The BELT model was considered due to the limitation of BERT of only considering 510 tokens. The basis of the model is the same BERT model as previously described. While other model architectures exist which allow for longer sequences, they are not as easily accessible for domain fine-tuning as exists for BERT. BELT extends BERT by adding a rolling window adding more sequences of BERT analyses which all are mean pooled at the end. Due to the size of the text corpora only a subset of the entire length of the messages were used – five windows of 510 tokens each, this captured the total length of the messages for 44 % of the patients. These five windows were split with two windows in the beginning of the text (0-1020 tokens) and three at the end (1530 last tokens). The rolling window means that for the BELT model five BERT models were stacked, the first model considering the first 510 tokens, the second 511-1020 etc.

**Fig. 1:**
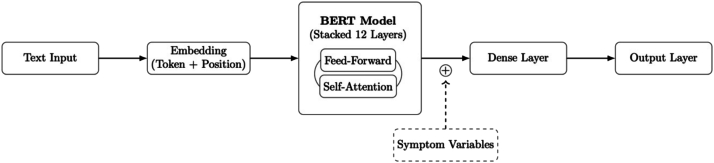
BERT model including symptoms architecture. For the BELT model this architecture was stacked five times with each model considering a different text window. BERT; Bidirectional Encoder Representations from transformers, BELT; BERT for Longer Text.

For each of the NLP models (TF-IDF, BERT, BELT) an additional model was trained by adding the symptom variables considered by the LR model (ergo symptoms and treatment indicator). For the TF-IDF model, this consisted of concatenating the sparse matrix representation of the word frequencies with the symptom variables, and training an Elastic Net regressor to optimise for the sparse matrix created. For the BERT model this consisted of creating a new classification head consisting of one dense layer, a dropout layer and a linear output layer, see Fig. 1 for model overview. The hidden layer concatenated the 768-dimensional matrix coming from the BERT model with the *sym*ptom variables (BERT-sym) (creating a 777-dimensional matrix) and was subsequently passed to the dropout layer (dropout rate = 0.1) and then to the linear output layer, these were included in the model training. The construction of the BELT model with *sym*ptom variables (BELT-sym) followed the same procedure as the BERT model (see Fig. 1), where each sub-model had a new classification head consisting of a hidden layer which concatenated the 768-dimensional vector with the symptom variables, a dropout layer and finally a linear output layer.

### 3.5 Hyperparameters

For each model we tuned the following hyperparameters inside the nested cross validation loop. For TF-IDF we considered number of features (500,5000), unigram and bigrams, and the Elastic Net considered an alpha of [3,1,0.5,0.2] and an l1 ratio of [0.2,0.5,0.8]. The BERT and BELT models had a batch size of 32 and 8 respectively, with 4 and 5 epochs respectively. The batch size was smaller in the BELT model due to the higher demand in memory of loading the larger chunks of texts, to counteract this we tried to increase epochs for the BELT model and modify the learning rate. The BERT models were optimized for a learning rate of [5e-5,2e-5], but due to computational time the BELT models learning rate was set to 2e-5, a smaller learning rate to reflect a smaller batch size. Generally, a low number of hyperparameters were considered due to the exponential growth in computing time due to the imputation x nested cross validation procedure.

### 3.6 Imputation

Imputation of missing data was done before cross-validation as proposed by (Jaeger et al., 2020). It was carried out in accordance with a multilevel imputation (Grund et al., 2018) with three imputations using MICE in R (van Buuren and Groothuis-Oudshoorn, 2011). A small number of datasets were selected due to exponentially growing computational requirements. The imputation was conducted with a linear mixed model with predictive mean matching (Van Buuren, 2018). Rubin's rules were used to combine estimations from the datasets (Van Buuren, 2018), this included the modified standard errors and degrees of freedom of the mean prediction across imputation sets to correct for the variance due to the imputation. Comparisons between models (including Welch's *t*-test) were done based on these means and standard errors.

### 3.7 Validation

We used nested cross-validation (NCV) based on results in (Poldrack et al., 2020) as well as our previous research (Hentati Isacsson et al., 2024a). A nested CV procedure in conjunction with multiple imputation improves the validity of the confidence intervals (Bates et al., 2021). To prevent overfitting, all hyperparameters were tuned in the inner cross-validation loop (Bates et al., 2021; Cawley and Talbot, 2017). The outer CV-loop consists of ten splits, and the inner of five. Each of the three imputed datasets went through the 10 × 5 CV loops. The inner CV loop determined the hyperparameter tuning, while the outer CV loops were used to compare the model performances.

### 3.8 Prediction metrics

Models were optimized for their Root Mean Squared Error (RMSE) score. Based on the scaling of the symptoms 0–1 the RMSE can be interpreted as the mean percentage wrong in the prediction. An RMSE of 0.1 would equal on average 10 % points from the true outcome in the prediction of the continuous outcome score. Balanced Accuracy (BACC) was also calculated to enable comparison to other models in the field as was F_1_ score. A BACC of 50 % represents a model not superior to guessing the majority category each time and an F_1_ score of 0.5 would represent a model randomly guessing the outcome.

## 4 Results

**Fig. 2:**
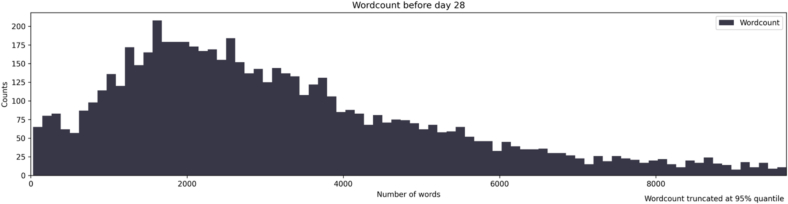
Histogram of wordcounts. Number of words sent in the patient-therapist exchange during the first 28 days of treatment.

The mean number of words between therapist and patient including homework formulations and answers were 3194 (SD = 2053) before day 28 in treatment, see Fig. 2 for the distribution. Except for an increase in the number of messages sent on treatment start there was no pattern over the treatment duration of when messages were sent. See Table 1 for baseline patient characteristics, for further details see (Hentati Isacsson et al., 2024a). Missing data for symptom measures ranged between 3 and 40 %, see supplement for imputation diagnostics and number of imputed values. For the complete code and several evaluative metrics of predictive performance please see the supplement.

**Table 1:** Baseline characteristics.

Characteristics	Sex	Age (mean (SD))	Education (count (%))^a^	Main Symptom (PRE)^b^ (mean (SD))	Main Symptom (POST)^b^ (mean (SD))
	Female: 4097 (62 %) Male: 2514 (38 %)	35 (11)	Primary: 504 (8 %) Secondary: 3121 (47 %) Postsecondary: 2782 (42 %)	44 % (15 %)	27 % (18 %)

a) *n* = 206 had missing on education b) Main symptom are the primary symptom measure for each treatment before treatment start (pre-treatment) and after treatment completion (post-treatment) scaled as a percent of maximum possible score. Panic Disorder Symptom Scale-Self Report, Montgomery-Åsberg Depression Rating Scale Self-report, and Leibowitz Social Anxiety Scale-Self report, respectively. Min was 0 for all three questionnaires and max was: 28, 54, 144 for PDSS-SR, MADRS-S, LSAS-SR respectively. Thus 44 % would be in raw points: 12.32, 23.76 and 63.36 for each scale respectively.

**Fig. 3:**
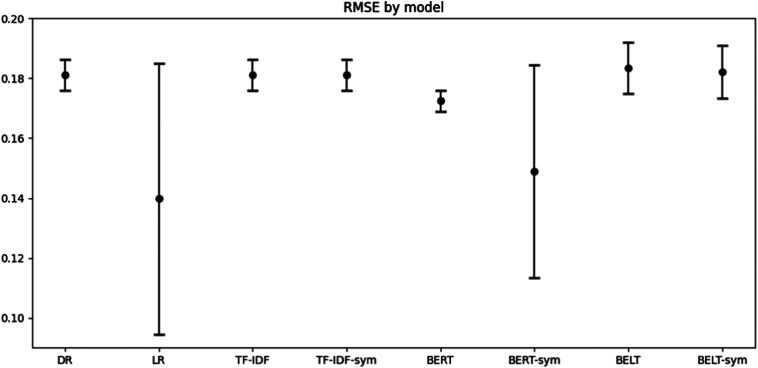
Root mean squared error for predicting symptom outcome. The root mean squared error (RMSE) mean and 95 % CI based on the 3 imputed datasets. The RMSE can be interpreted as the mean percentage wrong in the prediction. DR; Dummy Regressor, LR; Linear Regression; TF-IDF, Term Frequency-Inverse Document Frequency, BERT; Bidirectional Encoder Representations from transformers, BELT; BERT for Longer Text. Sym; Indicate that the model also used *sym*ptom variables.

**Fig. 4:**
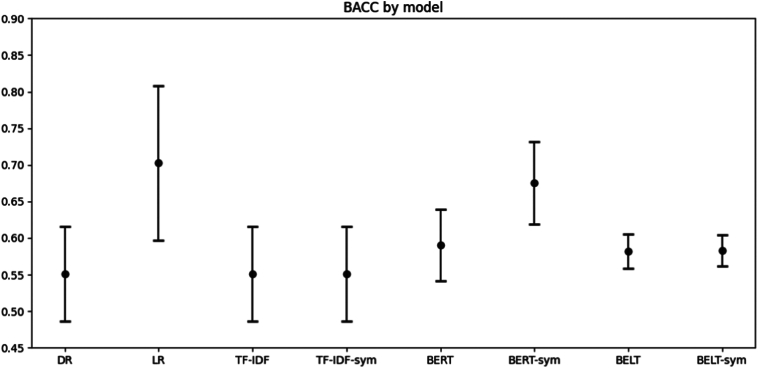
Prediction balanced accuracy for predicting symptom outcome. The Balanced Accuracy (BACC) mean and 95 % CI based on the 3 imputed datasets. DR; Dummy Regressor, LR; Linear Regression; TF-IDF, Term Frequency-Inverse Document Frequency, BERT; Bidirectional Encoder Representations from transformers, BELT; BERT for Longer Text. Sym; Indicate that the model also used *sym*ptom variables.

The models show some differences in performance. Fig. 3, Fig. 4 shows the Rubin's rules aggregated mean and 95 % confidence intervals for RMSE and BACC respectively, for each of the models. We found that only the BERT model with an RMSE of 0.17 [95 % CI: 0.1691, 0.1761], has a better RMSE score than the null model of Dummy Regressor (DR) which has 0.18 [0.1761, 0.1863]; t(77.88) = 2.80, *p* = 0.0065. When we look at BACC, both Linear Regression (LR) and BERT with added *sym*ptoms (BERT-sym) performed better than DR which has an BACC of 55 % [95 % CI: 48.68 %, 61.57 %]. Specifically, LR has a significant higher BACC of 70 % [95 % CI: 59.68 %, 80.85 %], t(3.99) = −4.77, *p* = 0.0089, as did BERT-sym with a BACC of 68 % [95 % CI: 61.93, 73.11 %], t(4.84) = −5.19, *p* = 0.0038.

The best point estimate for both RMSE (0.14 [95 % CI: 0.0944, 0.1852]) and BACC (70 % [95 % CI: 59.68 %, 80.85 %]) was from the LR model which did not use patient texts. LR had some significant differences to the other models. For RMSE it only was statistically significantly better to BELT (t(2.72) = −3.83, *p* = 0.0374) and BELT-*sym* (t(2.64) = −3.73, *p* = 0.0416). But for BACC it was better than DR (t(3.99) = −4.77, *p* = 0.0089), TF-IDF (t(3.99) = 4.77, p = 0.0089), TF-IDF-sym (t(3.99) = 4.77, p = 0.0089), BERT (t(3.29) = 3.83, *p* = 0.0265), BELT (t(2.79) = 4.36, *p* = 0.0260) and BELT-sym (t(2.79) = 4.34, *p* = 0.0261). Thus, it is unclear if any NLP model (even with symptom variables) brings added value for prediction beyond only using a model with symptom variables.

Adding symptoms to the model significantly improved only the BERT model, and then only for the BACC metric. The improvement was 8 %, t(4.89) = −4.13, *p* = 0.0095, for RMSE it was 0.02 lower, t(2.26) = 2.70, *p* = 0.1001. To facilitate comparison with other studies, the best models, LR, BERT and BERT-sym, achieve an F_1_-score of 0.66 [95 % CI: 0.4851, 0.8438], 0.55 [95 % CI: 0.5362, 0.5676], and 0.62 [95 % CI: 0.5015, 0.7401], respectively.

## 5 Discussion

The LR model had the best mean predictive performance for both RMSE and BACC. Of the NLP models only the BERT model outperformed a null model, as well as was the only model which improved when adding symptom variables, however only for BACC and not for the RMSE metric. While this improvement was significant (BERT vs BERT-sym) it is unclear whether BERT-sym was better than only considering symptom variables in the LR model. However, this indicates that a small signal on a relatively short amount of text is picked up with BERT, which is in line with previous research. The discrepancy between the mean performances of models were small; a difference of 0.02–0.04 in RMSE corresponds to a 2–4 % difference on the symptom scale sum score. For example, this would translate into a 1–2 point difference on the MADRS-S scale which would be considered a minimal difference and not clinically meaningful. The F_1_ score is on par with (Burkhardt et al., 2022) which had an F_1_ of 0.52 on a sample of 6500. While our results were below the F_1_ score of 0.73 found with text categorisations that was achieved with a sample size of 69 (Hoogendoorn et al., 2017). Adding the *sym*ptoms the BERT-sym F_1_ score of 0.62 is closer to the 0.69 found by (Gogoulou et al., 2021) with TF-IDF, however in that study the text used was closer to the timepoint of the outcome, the outcome definition was different, and the data filtered based on no missing data. Furthermore, Gogoulou et al., used a different embedding procedure and classifier, which could have improved their results. Our results indicate that the EN model had difficulty picking up a signal from the TF-IDF models sparse matrices, especially true when adding the 9 *sym*ptom vectors to a pre-existing matrix of 500.

Even if the benchmark model of LR had around the same point estimate as previous research (Hentati Isacsson et al., 2024a; Hentati Isacsson et al., 2024b) the large confidence intervals led to uncertainty. We see the same large confidence interval around BERT-sym, and this is due to the variation of prediction accuracy across the imputed dataset. A previous study with similar set up and only 3 imputed dataset found far narrower confidence intervals (Hentati Isacsson et al., 2024b), and so we believe this is most likely due to random variation in the imputation procedure because of the small number of imputed datasets. The amount of uncertainty was less pronounced for the BACC metric. This is likely due to chance having the point prediction fall on the favourable side of the dichotomisation cut off. This is supported by the fact that even the null model does not perform at precisely 50 % as expected when evaluated with BACC. Still, this indicates that, past *sym*ptoms are a stronger predictor of post-treatment symptoms than the text-based interaction between patient and therapist.

Contrary to expectation the BERT model outperformed the BELT models, albeit for the RMSE metric the BERT-sym model showed too large uncertainty to draw conclusions. It is possible that stacking BERT models which used a larger scope of text, with smaller batch size (due to memory requirements), increased the uncertainty and made the ensemble mean harder to converge properly. This despite our efforts to raise the number of epochs and tuning for a smaller learning rate. Furthermore, this uncertainty could have made it hard for the additional 9 features from the symptom addition to impact the predictions.

We believe several factors influences why the text signal was weak. Our first concern is that the text dataset was too small, with the limited number of patient-therapist interactions and limited computational power the transformer network for BERT and BELT had a hard time fine tuning to the predictive target. Secondly, a larger text window of the therapeutic interaction could also limit the predictive capabilities, ergo what is said during the first few weeks of treatment may not reflect the final outcome or the treatment progress made during later weeks. Thirdly, in line with the above a possible way to ameliorate this would be to feature engineer the text data, disentangling the therapist-patient exchange, and getting clinical insight from experts on what parts of the text data to focus on.

## 6 Limitations

While we used multiple imputation to mitigate the bias of single-pass imputation (Grund et al., 2018), our small numbers likely influenced our estimates, reducing the precision of our models. A larger number of imputed datasets would have strengthened our conclusions (e.g. ≥10). However, this was avoided due to the exponential increase in computational time. The computational complexity was also the reason we explored such a few numbers of hyperparameters, and it is likely a better precision could've been reached by exploring further. This seems especially likely with the BELT model, which was also hampered by memory limitation of batch sizes and could've benefited from exploring different window-splitting strategies. Furthermore, we could also have explored other ways to handle the symptom variables, e.g. had a linear dense layer for TF-IDF or added several layers to BERT and BELT. While many of these limitations stem from the decision to use multiple imputation we believe that this imputation also increase the reliability of our findings.

One potential influencing factor is that models considered the therapeutic exchange between both patient and therapist, future models could focus on one part of the conversation to see if that bears more predictive value or use time-dependent models. Furthermore, future studies could focus on specific parts of the text from the specific treatments to investigate the predictive value in expert-picked message exchanges between therapist and patient. Due to the overall low number of patients for an NLP context expert-picked variables or message-exchanges could help the models as has been shown for other variables in (Hentati Isacsson et al., 2024a). Finally, the fine-tuning of BERT and BELT would likely have been more effective with a larger sample size. However, due to the sensitive nature of the dataset, all analyses were conducted within a clinical environment, which further restricted the computational resources available for more extensive hyperparameter tuning.

While the topic of the present study was the predictive capabilities of NLP models in a clinical context, we believe that this context could be further understood if future models also include specific language features to understand predictions better. Such investigations could focus on linguistic features (such as emotional tone) or predictive phrases. This is especially true for this clinical context where clinicians might be interested in what phrases or language features inform predictions. Furthermore, unsupervised text exploration could be suitable to explore possible patterns in the data without the need for labels, such as topic modelling or sentiment trajectories over the course of treatment. Due to the minimal differences found in this paper, such future work on unsupervised models could help understand the text data in internet delivered treatment further before future supervised prediction work. In the future such work could help clinicians by flagging certain messages for clinician review or monitor other outcomes than symptom outcome.

While these predictive models are based on clinical dataset with high fidelity, the current limitations and minimal predictive capabilities underscore the need for further research before implementations in clinical settings. Currently minimal models using symptoms and other clinical variables are a more feasible target for study such as in (Bjurner et al., 2025) where all predictions are handled by a clinician. Additionally, implementations carry with them their own set of challenges and ethical considerations beyond the current scope (Löchner et al., 2025; Svensson et al., 2025). Furthermore, generalizations are impacted since this is a single-site study, and further pooling of data from different clinics could help both with boosting the sample size and generalizability.

## 7 Conclusion

Our result support that patient-therapist text can be predictive of post-treatment symptom outcome. Based on our results, the predictive capabilities seem small and the relation tenues between a text-excerpt between patient-therapist content and a self-rated symptom several weeks later. Furthermore, training a model to consider both the patient-therapist text as well as the concurrent self-rated symptom can strengthen the prediction but possibly not beyond only using the symptom variables. These results contribute to the limited literature on using patient-therapist text in ICBT to predict self-rated post-treatment symptom outcomes. While the patient-therapist conversation is one part of the treatment, its relevance for prediction remains difficult to assess. Language models have many potential applications beyond prospective prediction. Future research on patient-therapist conversations could explore avenues such as automatic goal formulation and functional analysis of treatment behaviours.

## Funding

This work was mainly supported by The 10.13039/501100004359Swedish Research Council (VR), 10.13039/100007436The Erling Persson family foundation (EP-Stiftelsen), and The Swedish ALF-agreement between the Swedish government and the county councils, with additional funding by the 10.13039/501100001729Swedish Foundation for Strategic Research (SSF). The funding sources were not involved in any part of the study.

## Declaration of competing interest

The authors declare that they have no known competing financial interests or personal relationships that could have appeared to influence the work reported in this paper.

## Appendix A Supplementary data

Supplementary material 1 Image 1

Supplementary material 2 Image 2
