# Supplementary material for: Natural language processing models for predicting treatment outcomes in internet-delivered cognitive behavioral therapy
Source: Internet Interv. 2025 Oct 10;42:100879. doi: 10.1016/j.invent.2025.100879 (PMC12547759; doi:10.1016/j.invent.2025.100879)
Supplement: Supplementary file 2 — Supplementary material 2 [file mmc2.docx]

Supplement document:

**Manuscript**

Natural Language Processing Models for Predicting Treatment Outcomes in Internet-delivered Cognitive Behavioural Therapy

**Note**

This supplement contains more details for the main paper, including link to raw data, elaboration on the method section, additional figures and results.

The entire code for prediction, model tuning and result processing can be found at: https://osf.io/dzfw7/?view_only=da972177788a4c3d8a6e6780c276886e

**Contents**

**Symptom outcome**

**Imputation diagnostics and number of imputed values**

Table 1

**Supplementary files**

Following files found at: https://osf.io/dzfw7/?view_only=da972177788a4c3d8a6e6780c276886e

1. Code (rar file)

2. Results all metrics (csv file)

Symptom outcome

After the continuous prediction was made, the score was dichotomized into ‘success’ if the predicted score was either 1) below the corresponding questionnaire’s cut-off for remission or 2) a 50% reduction of symptoms from treatment start. See (Hentati Isacsson, Zantvoort, et al., 2024) for a complete description.

**Imputation diagnostics and number of imputed values**

| Table 1. Imputed values | | | | |
| --- | --- | --- | --- | --- |
| Variable | N percent | n | influx | outflux |
| MADRS-S screening | 0,97 | 6511 | 0,02 | 0,94 |
| LSAS-SR screening | 0,90 | 6013 | 0,08 | 0,80 |
| PDSS-SR screening | 0,93 | 6242 | 0,05 | 0,85 |
| Mainsymptom Pre-treatment | 0,96 | 6441 | 0,02 | 0,90 |
| Mainsymptom Week 1 | 0,82 | 5491 | 0,12 | 0,58 |
| Mainsymptom Week 2 | 0,84 | 5642 | 0,09 | 0,59 |
| Mainsymptom Week 3 | 0,83 | 5538 | 0,10 | 0,55 |
| Mainsymptom Week 4 | 0,81 | 5408 | 0,11 | 0,50 |
| Mainsymptom Week 5 | 0,77 | 5146 | 0,14 | 0,42 |
| Mainsymptom Week 6 | 0,74 | 4951 | 0,17 | 0,37 |
| Mainsymptom Week 7 | 0,70 | 4707 | 0,20 | 0,31 |
| Mainsymptom Week 8 | 0,67 | 4509 | 0,22 | 0,27 |
| Mainsymptom Week 9 | 0,64 | 4282 | 0,26 | 0,24 |
| Mainsymptom Week 10 | 0,60 | 4037 | 0,30 | 0,21 |
| Mainsymptom Post-treatment* | 0,76 | 5089 | 0,16 | 0,46 |
| *Post-treatment is the outcome  MADRS-S; Montgomery-Åsberg Depression Rating Scale Self-report, LSAS-SR; Leibowitz Social Anxiety Scale-Self report, PDSS-SR; Panic Disorder Symptom Scale-Self Report. Influx; Influx depends on the amount of missing, it is 0 for complete data, if the amount of missing is equal the variable with higher influx is better connected to observed data. Outflux; Outflux depends on also on the amount of missing, it is 1 for complete data, if the amount of missing is equal the variable with a higher outflux is better connected to missing data and more useful for imputing. | | | | |

References

Hentati Isacsson, N., Ben Abdesslem, F., Forsell, E., Boman, M., & Kaldo, V. (2024). Methodological choices and clinical usefulness for machine learning predictions of outcome in Internet-based cognitive behavioural therapy. *Communications Medicine*, *4*(1), 1–11. https://doi.org/10.1038/s43856-024-00626-4

Hentati Isacsson, N., Zantvoort, K., Forsell, E., Boman, M., & Kaldo, V. (2024). Making the most out of timeseries symptom data: A machine learning study on symptom predictions of internet-based CBT. *Internet Interventions*, *38*, 100773. https://doi.org/10.1016/j.invent.2024.100773
